# Brugadaphobia

**DOI:** 10.19102/icrm.2025.16091

**Published:** 2025-09-15

**Authors:** Laszlo Littmann, William C. Bock

**Affiliations:** 1 Department of Internal Medicine, Atrium Health Carolinas Medical Center, Charlotte, NC, USA; 2 Atrium Health Sanger Heart and Vascular Institute, Charlotte, NC, USA

**Keywords:** Brugada phenocopy, Brugada syndrome, implantable cardioverter-defibrillator, sudden cardiac death

## Abstract

We present the case of a young man with cocaine intoxication whose electrocardiogram (ECG) on presentation showed a typical type 1 Brugada pattern. The patient had no personal or family history of unexplained syncope or sudden cardiac death. The ECG quickly normalized, and follow-up ECGs continued to be normal. Nevertheless, the patient and family members insisted on implantation of a cardioverter-defibrillator. The purpose of this case report is to discuss the somewhat unfounded fear of sudden cardiac death of asymptomatic patients with Brugada-type ECGs that has been termed “brugadaphobia” and to highlight the difficult and controversial decision-making process that should include discussions about the possible benefits and harms of an overly active diagnostic and therapeutic approach.

There is a general consensus that patients with a spontaneously occurring type 1 Brugada pattern in the electrocardiogram (ECG), especially if they had experienced syncope, ventricular tachycardia, resuscitated sudden death, or nocturnal agonal respiration, should be offered implantation of an implantable cardioverter-defibrillator (ICD).^1^ Treatment recommendations are more fluid for asymptomatic patients; for those with a type 2 Brugada pattern; and in patients in whom the Brugada ECG did not appear spontaneously but was provoked by various conditions, including drug challenge. The results of several large-scale studies help in the risk stratification of such controversial cases.^2–5^ Clinical experience, however, has shown that, frequently, even those patients with a very low objective risk of sudden death continue to be fearful and insist on ICD implantation. This somewhat irrational fear of sudden death, which can be fueled by conflicting recommendations, has recently been termed “brugadaphobia.”^6,7^ We present the case of a brugadaphobic patient who underwent probably unnecessary ICD implantation and then experienced complications from the procedure.

## Case presentation

**Figure 1:**
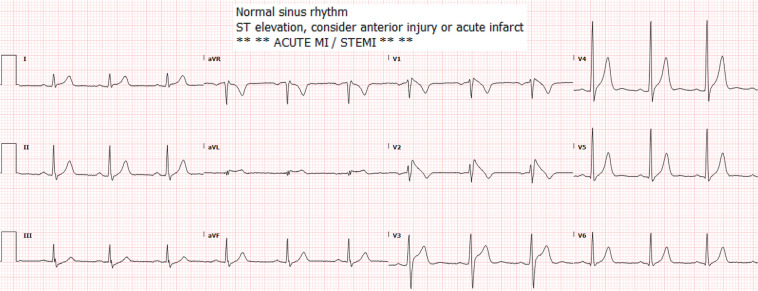
Twelve-lead electrocardiogram on admission. Software interpretation was enlarged for legibility. *Abbreviations:* MI, myocardial infarction; STEMI, ST-segment–elevation myocardial infarction.

A previously healthy 30-year-old Caucasian man was hospitalized because of confusion and extreme sleepiness. His relatives reported heavy cocaine use the day before. The patient had been groggy the whole day. During brief periods when he was alert, the patient complained of mild left-sided chest pain. On admission, the patient was somnolent but in no distress, not diaphoretic, and did not appear to be dyspneic. The physical examination findings were unremarkable. His core temperature was 99°F, with a heart rate of 75 bpm and blood pressure of 125/68 mmHg. The point-of-care troponin I level was 0.40 ng/mL (upper limit of normal, 0.04 ng/mL). The ECG showed sinus rhythm with a 2–5-mm high take-off upward convex ST elevation followed by negative T-waves in leads V1 and V2 with an upward convex ST elevation in V3, typical of a type 1 Brugada pattern.^1^ The interpretation software indicated an anterior injury and an acute infarct (ST-segment–elevation myocardial infarction [STEMI]) **(Figure 1)**. The Brugada ECG is frequently misread by computerized software as STEMI.^8^ It is easy to distinguish the two by the fact that, in STEMI, unlike Brugada, one can almost always see ST depression in mirror-image leads.^8^ Here, there was no such depression.

**Figure 2:**
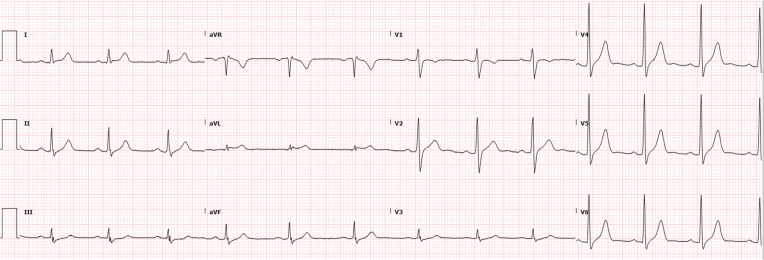
Electrocardiogram recorded 1 day later.

The Brugada ECG was recognized, and it was felt that the clinical picture was not suggestive of STEMI. A bedside echocardiogram did not show wall motion abnormality. The patient’s mental status gradually cleared up. He confirmed that he had no family history of sudden death and no personal history of syncope. Troponin I peaked at 0.90 ng/mL; such modest troponin elevations are frequently seen in cocaine intoxication. The following day, his ECG returned to normal **(Figure 2)**. Lead placement in the two ECGs may have been slightly different, but an ECG from a year before and subsequent ECGs were also normal. The risk of drug abuse was discussed, and it was strongly recommended that he avoid drugs that can provoke Brugada. We did not feel that either further testing or ICD implantation was indicated. The patient was discharged without medication.

One year later, the patient and his girlfriend presented to the cardiac clinic requesting implantation of a defibrillator. The girlfriend had read up extensively about Brugada syndrome on the internet and was concerned about her young, healthy partner dying suddenly. We felt that the patient’s and his girlfriend’s concerns were exaggerated, but admitted the uncertainties regarding this issue. We offered electrophysiologic testing for further risk stratification but acknowledged that this too was controversial and of questionable benefit. The patient declined further diagnostic testing, which made it difficult to differentiate true Brugada syndrome from concealed Brugada syndrome or Brugada phenocopy. The risks and benefits of ICD implantation were discussed. Although the cardiologist was not convinced, they felt obliged to implant an ICD.

Over a 3-year follow-up, the patient had no arrhythmic event or syncope, and ICD interrogation did not show any dysrhythmia. Following ICD implantation, however, the patient had numerous complaints of pain and discomfort at the ICD site. Eventually, he required a surgical ICD pocket revision for refractory symptoms.

## Discussion

Brugada syndrome is an inherited genetic disease frequently affecting young men.^1^ Despite not having demonstrable structural heart disease, patients with Brugada syndrome are at risk of sudden death that may occur during sleep. The most reliable intervention to prevent arrhythmic death is an ICD.^1^ The primary electrocardiographic risk of sudden death is a documented spontaneous (unprovoked) type 1 Brugada ECG. Clinical markers of increased risk include a history of unexplained syncope, nocturnal agonal respiration, resuscitated sudden death, ventricular tachycardia, and possibly a family history of unexplained sudden death at a young age.^1^

Over the last several decades, it has become clear that a Brugada-type ECG abnormality can be present in patients who may not have the genetic disease. It can be provoked by metabolic abnormalities, medications, drugs including cocaine,^9^ mechanical compression of the right ventricle, pericardial disease, pulmonary embolism, and ischemia. Conditions where the Brugada ECG was provoked by one of the listed conditions are termed Brugada sign, Brugada phenocopy, or acquired Brugada syndrome.^1,10,11^ In addition to manifest Brugada syndrome and Brugada phenocopy, some consider a third category, termed “concealed Brugada syndrome,” to describe cases where the Brugada ECG was exposed by unmasking agents such as sodium channel blockers. Toxic levels of cocaine have relatively strong sodium channel–blocking effects.^1,9,11^ Some experts believe that concealed Brugada syndrome, especially when associated with a family history of syncope and/or sudden death, signifies an increased risk of cardiac arrhythmia and sudden death.^11^ Regardless of the terminology and category assignment, however, those patients with drug-induced Brugada ECG who have no documentation of a spontaneous Brugada pattern and who have not had Brugada-related symptoms are at a very low risk of sudden death. In a study of 45 patients who only had drug-induced Brugada abnormality, such as our patient, there was no arrhythmic event over a median follow-up of 8.1 years.^2^ In another study of 111 low-risk patients followed up for an average of 4.6 years, there was no incidence of sudden death.^3^ In a cumulative study of 486 asymptomatic patients without a spontaneous Brugada pattern, again, there was no sudden death noted.^4^ In a recent study of 610 asymptomatic patients with drug-induced Brugada, there was one case of sudden death over a median follow-up of 6 years (0.03% per year), and that patient did not comply with follow-up recommendations.^5^ While advocated for by some, the role of electrophysiology study, drug challenge, or genetic testing in the risk stratification of low-risk patients is highly questionable.^12–15^ Although a recent study suggested some value for electrophysiologic and genetic testing of patients with drug-induced Brugada, the real-life clinical value of the study is unclear.^16^ Of the 606 study patients, only eight patients (1.3%) without a history of unexplained syncope had a primary outcome event, which included appropriate ICD therapies, including antitachycardia pacing, but no sudden death. Seventy percent of asymptomatic patients with drug-induced Brugada did not receive an ICD, but none of them experienced sudden death.^16^

Patients and family members who are told of the Brugada abnormality frequently become very anxious about the risk of sudden death. Typically, as in the case presented, a female partner of a man searches the web where, obviously, nuances are not discussed. This somewhat irrational fear of sudden death fueled by the internet has been termed “brugadaphobia” by Dr. Viskin.^6,7^ Brugadaphobia poses a challenge to both patients and providers. On the one hand, as discussed, patients with no high-risk characteristics are very unlikely to suffer an arrhythmic death. On the other hand, the absence of an event observed in clinical studies involving a relatively small number of patients does not rule out a risk entirely.^17^ In the three quoted studies with no events, based on the number of patients enrolled, statistically, one could not rule out an up to 6.7%, 2.7%, or 0.6% chance of sudden death, respectively.^2–4,17^ The decision-making process should include discussions on the competing risks of no intervention versus the substantial complications associated with an ICD in a young patient, as well as patient preference and risk tolerance. Even when the physician feels otherwise, it may be impossible to deny an ICD requested by a brugadaphobic patient.

## Conclusions

The appropriate workup and management of patients with possible Brugada syndrome is highly controversial, even among authorities in the field. There are some who recommend a detailed workup to search for markers that would warrant implantation of an ICD. Many other experts, however, believe that, for those low-risk patients who have not had Brugada-associated symptoms and who have not had a spontaneous type 1 Brugada ECG recorded, additional testing may cause more harm than benefit. Patients who are searching for the appropriate approach may be caught in the middle. Our responsibility is to present all relevant facts, offer our best opinion, and then work together to come up with a plan that is not unreasonable and may be acceptable to all.
